# Assessing Culturally Relevant Variables in Predicting Science Outcomes in Asian American Kindergartners

**DOI:** 10.3390/bs16040550

**Published:** 2026-04-07

**Authors:** Josh Medrano, Dana Miller-Cotto

**Affiliations:** School of Education, University of California, Berkeley, CA 94720, USA; dmillerc@berkeley.edu

**Keywords:** science achievement, self-regulation, Asian American

## Abstract

Though separate research has found that early experiences, parental beliefs, and cognitive skills all influence science learning, science remains an underexamined domain compared to math and reading, despite its policy and societal implications. We integrate and expand on previous research by examining culturally relevant variables in different subgroups of Asian American kindergartners (*N* = 894). Guided by the Opportunity-Propensity Model of Achievement, we conducted a multi-group path analysis with science scores as the outcome, and propensity (self-regulation, social skills, and prior knowledge), opportunity (e.g., parent and child reading, TV-watching routine), and antecedent variables (e.g., poverty, SES, number of siblings and close grandparents, parental expectations, primary language at home, immigrant status) as predictors. We expected that propensity and opportunity variables would mediate the effects of antecedent variables. We conducted a multi-group path analysis, in which we examined differences between subgroups (China, India, Vietnam, Other East, Other Southeast, Other). Although we did not find heterogeneity in science achievement among subgroups, we found various direct and indirect effects at the subgroup level. Findings suggest that Asian American children may generally benefit from enhanced self-regulatory skills and prior knowledge, though some subgroups may benefit specifically from having fewer TV-watching rules and non-structured activities. We also recommend further disaggregation and reporting of data to better support learners.

## 1. Introduction

Science is a foundational domain of cognitive development, and early science skills predict later participation in science and STEM-related activities, underscoring the importance of supporting science learning in early childhood (Cabe Trundle & Saçkes, 2021). Yet existing research suggests that early science achievement is shaped by multiple contextual and cognitive factors, including maternal education (Guo et al., 2015), home language use (Chang & Shih, 2023), and other cognitive abilities (Nayfeld et al., 2013). Despite growing policy attention to science education, the empirical literature remains disproportionately focused on mathematics, limiting a comprehensive understanding of early STEM learning. This imbalance is notable given well-documented socioeconomic and racial disparities in early math achievement (Dotterer et al., 2012; Miller-Cotto et al., 2024), raising questions about whether similar patterns exist for science. Asian Americans are frequently characterized as a homogeneous “model minority” (Kao, 1995), despite substantial subgroup variation in academic outcomes (Fuligni, 1997), language use, socioeconomic status, and access to STEM-related opportunities (Budiman & Ruiz, 2021; Budiman, 2024; Loi, 2024). Recognizing this heterogeneity is essential for developing equitable policies and interventions. Using a nationally representative dataset, we examine predictors of science achievement across Asian American subgroups, guided by the Opportunity-Propensity Model of achievement (Byrnes, 2020), to clarify how early experiences, parental beliefs, and cognitive skills jointly shape early science outcomes.

### 1.1. The Opportunity-Propensity Model

The Opportunity-Propensity (O-P) model provides a way to integrate individual differences, environment, and socioeconomic or family background in predicting achievement outcomes (Byrnes & Miller, 2007). In a typical study, achievement score serves as the dependent variable, whereas variables of interest serve as independent variables, classified into three categories: propensities, opportunities, and antecedents. Propensity variables refer to child characteristics such as their prior knowledge, executive functioning, and social skills. Opportunity variables refer to characteristics of children’s home and classroom environments. Antecedent variables refer to macro-level factors such as SES, race, and cultural factors that may explain why children possess certain levels of opportunity and propensity variables (Byrnes & Wasik, 2009).

While the O-P model originally posited all variables as predictors (Byrnes & Miller, 2007), opportunity and propensity variables may also mediate the role of antecedent variables (Byrnes et al., 2018; Wang et al., 2013). Researchers have also examined untested factors, such as home literacy activities and motor skills (A. Ribner et al., 2019), primary language used at home (Byrnes et al., 2018), and cognitive stimulation (Byrnes et al., 2018). The value of this model lies in its flexibility to include variables, modify the relations between factors, and account for age. These inclusions and modifications have theoretical implications for the role of particular variables that are often understudied, such as culture; by proposing directional links between background, environment, and achievement, this framework thus helps in understanding how those variables might affect the development of academic skills.

The current study examines how children’s science achievement is influenced by their family background and environment. We examine the role of culture, which can be defined as a set of norms, ideas, values, conventions, and behaviors that are shared by a group of people (Bornstein, 2015). One’s culture may have values and behavior that are normative for them but not necessarily for another; there may also be factors that are more relevant for one culture than another (e.g., having multiple caregivers, speaking more than one language at home). In the current paper, we investigate the role of culture in the development of children’s science achievement through variables such as subgroup, immigration status, and number of siblings and close grandparents.

#### 1.1.1. Antecedents: Family Structure and Demographics

Antecedent variables refer to demographic and family-level factors in a child’s environment. These factors typically include socioeconomic status; disparities in achievement and cognitive abilities, as well as investments parents make in their children, have been found between different levels of socioeconomic status (Bradley & Corwyn, 2002; Duncan et al., 1998; Evans & Rosenbaum, 2008). Other factors are less studied but are more structural, fixed, and more culturally relevant, such as the number of relatives in the household, language spoken at home, parental expectations regarding their child’s educational attainment, and the immigration status of the parents and/or the child (Kruzik et al., 2024; McLoyd, 1998)—all of which can indirectly affect achievement and development (Davis-Kean, 2005). The present study examined how these factors contribute to the science achievement of Asian American kindergartners.

#### 1.1.2. Opportunities: Home Environment and Parenting Practices

Opportunity variables refer to the home environment, parenting practices, and the opportunities families have due to their background. Theoretical models have outlined and explained how family structures and demographic backgrounds (i.e., the antecedent variables) can influence achievement. For instance, the family stress model (Masarik & Conger, 2017) suggests that lower socioeconomic status and living in poverty lead to fewer resources (or challenges in accessing high-quality resources, such as extracurricular activities) and lower-quality parenting, which subsequently adversely affect achievement.

Conversely, the family investment model argues that a higher socioeconomic status provides those resources, which in turn enhance achievement (Davis-Kean, 2005). For example, in a sample of German preschoolers, science related activities were associated with science knowledge and mediated by parental education, home language, and parental interest in science (Junge et al., 2021). In another study with Asian American immigrant Kindergartners, parents’ literacy activities in English at time 1 were positively associated with children’s English literacy skills at that time (Xu et al., 2017). Additionally, parents’ literacy activities in both English and their native language at time 1 predicted children’s interest in literacy at time 2, which, in turn, correlated with children’s English oral language skills at time 2 (Xu et al., 2017). Other opportunity variables related to achievement may also include family routines (Selman & Dilworth-Bart, 2024) and extracurricular activities (e.g., museums, dance classes; Ren & Zhang, 2020; Kim, 2021). In this study, we examined whether such resources predict science achievement and mediate the effects of family background on science achievement in Asian American children. Although previous research has shown that these resources are essential in general, there may be differences in how and whether families use these resources across subgroups of Asian Americans (e.g., Gibbs et al., 2017; Medrano & Miller-Cotto, 2026).

#### 1.1.3. Propensities: Self-Regulation, Social Skills, and Prior Knowledge

Propensity variables refer to individuals’ internal characteristics that may facilitate achievement (Byrnes et al., 2018). One such skill, self-regulation, refers to the management of one’s thoughts, behaviors, and emotions, and encompasses constructs such as executive functions (the regulation of thoughts during tasks) and approaches to learning (the regulation of learning behaviors). Self-regulation, executive functions, and approaches to learning are strong predictors of achievement (Allan et al., 2014; Bustamante et al., 2017, 2018b; Nayfeld et al., 2013; Vitiello & Greenfield, 2017). For example, in a study with the same dataset, students with greater executive function at the start of kindergarten benefited more from mathematics instruction than those with lower executive function (A. D. Ribner, 2020). In another study, with Head Start children, approaches to learning had a bidirectional relationship with science performance, in that growth in either one led to growth in the other (Bustamante et al., 2018b).

Social skills—in this study, a composite of self-control, interpersonal skills, externalizing problem behaviors, and internalizing problem behaviors—also influence development. In a study in a previous iteration of the present dataset, those who had high achievement scores in math, science, and reading from kindergarten through fifth grade exhibited higher self-control and interpersonal skills and fewer externalizing and internalizing problem behaviors than those who had low achievement scores (Sung & Chang, 2010). Social skills may be critical as learners acquire knowledge through observation of others’ behaviors or interactions with adults or more knowledgeable peers (Vygotsky, 1978; Bandura, 1977).

In addition to these skills, prior knowledge—such as math and reading skills—contributes to achievement. Math skills such as measurement, comparing and contrasting, and sorting and organizing are often used in science (Bustamante et al., 2018a, Education Sciences). Studies have shown that early math skills predict later science achievement in preschool and kindergarten (Guo et al., 2015; Nayfeld et al., 2013; Westerberg et al., 2021). Similarly, children’s reading comprehension and linguistic abilities may contribute to science performance by enabling them to acquire science vocabulary and engage in science process skills such as planning, predicting, and drawing inferences (French, 2004). These process skills are domain-general skills that can be applied to any domain (Jirout & Zimmerman, 2015), although they may be more common in science classrooms, as opposed to mathematical or reading classrooms. They may develop prior to any formal instruction, as children explore the world around them, be curious and ask questions—for example, they may classify a novel item and ask themselves or more knowledgeable individuals whether it fits any of their existing schema. These skills may develop spontaneously or through instruction (Jirout & Zimmerman, 2015). Scientific thinking, itself, however, uses these process skills along with other skills and knowledge that are related to mathematical thinking and reading.

In the current study, we examine the role of propensity variables as mediators. For example, self-regulation is shaped by the family, home, and school environments (see Blair & Raver, 2015, for a review)—poverty, lower socioeconomic status, and related conditions (e.g., caregiver resources) negatively affect the development of self-regulation, hindering achievement (Blair & Raver, 2015). In another example, cultural practices and background may influence children’s social skills, which may affect their scientific reasoning (Callanan et al., 2020; Lombardi et al., 2024).

In summary, we used the O-P model to examine the effects of different predictors on science achievement. Our paper examines this model in the context of Asian American families. We extend prior work on Asian American development by including culturally relevant variables related to the family structure (number of close grandparents and siblings), primary language spoken at home, parental expectations, and immigration status (Medrano & Miller-Cotto, 2026). The model has several policy implications. For instance, policymakers and educators may choose to provide greater opportunities to improve executive functions for elementary school students. (For a review of the development of the O-P model and the model’s implications, see Byrnes (2020)). In this study, we investigated how the O-P model explains academic outcomes for different groups of Asian American children and whether the significant direct effects and mediators are the same for all subgroups.

### 1.2. The Current Study

**Figure 1:**
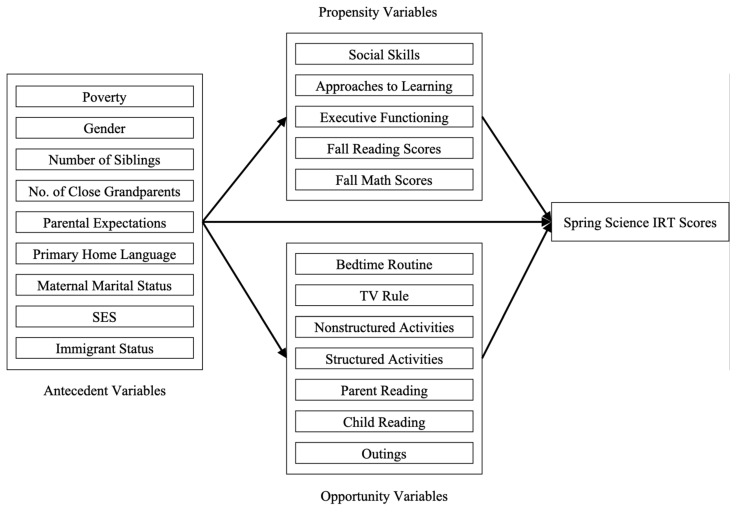
Proposed model. Antecedent, propensity, and opportunity variables directly influence spring science scores. Propensity and opportunity variables also mediate the effects of antecedent variables.

We sought to determine how antecedent, opportunity, and propensity variables predict science achievement in Asian American children (see Figure 1). Emerging research suggests that some variables (e.g., family background, parental expectations) are more influential than others (e.g., extracurricular activities and parenting practices) in the achievements of certain groups. For example, in a study with a similar sample, the indirect effects observed at the overall group level were absent in the Vietnamese and Other Southeast Asian subgroups (Medrano & Miller-Cotto, 2026).

As in Medrano and Miller-Cotto (2026), we focused on antecedent variables that may be specific to Asian Americans or minoritized groups in general. We deem these variables culturally relevant, in that they are potentially relevant to all Asian Americans; however, the extent to which they are relevant may differ by subgroup. For instance, a core value for Asian Americans is familism, which refers to “prioritizing one’s family over oneself” (Schwartz, 2007, p. 101). In a study with Asian American adolescents, family obligation buffered the adverse effects of socioeconomic stress, helping maintain high educational expectations, aspirations, and attitudes toward academic success (Kiang et al., 2013). Some evidence suggests that subgroups may not exhibit this value or are influenced by it to the same extent. For example, in a measurement validity study, Filipino Americans exhibited a stronger sense of familism than Korean Americans, although both subgroups generally uphold familism (Choi et al., 2021). Based on previous findings regarding the impact of familism on success, we anticipate that family structure (operationalized here as the number of siblings and close grandparents) will positively contribute to achievement. However, we have no specific predictions concerning particular subgroups.

The second variable of interest is immigration status. Earlier-generation immigrants (persons who were or who have parents who were born outside of the U.S.) have been shown to outperform later-generation immigrants in academics (see Duong et al., 2016, for a meta-analysis; Fuligni, 1997). In the current study, we defined immigrants as children who were born outside the U.S. or whose parents were born outside the U.S. Although the Asian American population has increased overall since 1965, other factors may also affect immigration status (Ngo & Lee, 2007). Importantly, some immigrants moved to the U.S. due to war and geopolitical conflicts; immigrants from such backgrounds—though not specified here—may begin with lower income, which can affect their achievements (Rahman & Paik, 2017).

The third variable is the primary language at home. Those who speak their native language or more at home would be considered bilingual or multilingual. Though their relations are more nuanced, bilingualism and multilingualism have been associated with greater cognitive abilities (Langeloo et al., 2019; van den Noort et al., 2019). The final variable of interest is parental expectations. These expectations may influence children’s outcomes through parenting behaviors (Sy & Schulenberg, 2005), exposure to supplementary education, such as tutoring (Zhou & Kim, 2006), and other activities outside the home (Kim, 2021). However, differences in resources by socioeconomic status, immigration status, and reasons for immigration may lead to subgroup differences in how expectations affect children’s outcomes. For example, Rahman and Paik (2017) showed, in their historical analysis, even though expectations might be high for all South Asians subgroups, in addition to the model minority stereotyping of all South Asians as highly achieving, that some subgroups—particularly from Bangladesh or Pakistan—might not have assistance or capital that they need to achieve those outcomes due to poverty and lack of government attention.

Our research questions were: (1) What is the diversity of science achievement among Asian American children, and (2) what factors predict this diversity? We predicted that the antecedent factors above—presence of grandparents and siblings, immigration status, primary language at home, and parental expectations—will both directly and indirectly affect math achievement, and that there are subgroup differences. We also predicted that opportunity and propensity variables would mediate these effects at the overall and subgroup levels.

## 2. Materials and Methods

### 2.1. Sample

This study focused on a subset of Asian American children entering kindergarten for the first time in the fall of 2010. These children came from the Early Childhood Longitudinal Study, Kindergarten Cohort-2011 (ECLS-K: 2011), a study sponsored by the Institute of Education Sciences. The study follows a complex study design. A nationally representative sample was recruited using a three-stage probability sampling method that included stratified, proportional, and cluster sampling strategies. The full dataset was consisted of 18,200 kindergartners (rounded to the nearest 50 in accordance with the restricted data use license). Students were enrolled in 968 public and private schools across all 50 states, with 23 kindergarten children randomly selected from each school for the study.

Within the dataset, 1211 were identified as “Asian Americans.” However, we were only able to extrapolate the subgroups of 962 children (79%) from their parents’ birth countries (841), primary languages spoken at home (71), and the children’s birth countries (50). Based on the outcome variable, spring science scores, listwise deletion was performed for a final sample of 894 children.

Six subgroups were created such that they are proportionally close to each other in terms of sample size, with 56 countries represented overall. Only Vietnam, China, and India had more than 100 representatives each; the Other East and Other Southeast regions were created based on geographical regions and were made up of at least 100 representatives each; the Other subgroup included other countries and regions (see Table 1). The Other East subgroup represented countries like South Korea (*n* = 53), Taiwan (*n* = 22), and Japan (*n* = 12); the Other Southeast subgroup represented the Philippines (*n* = 80) and Thailand (*n* = 20), among others; the Other subgroup represented South Asian countries like Pakistan (*n* = 30) and Bangladesh (*n* = 14), as well as those born in non-Asian countries, such as the United States (*n* = 57), Venezuela (*n* = 4), and American Samoa (*n* = 3), but were otherwise coded as Asian American. These non-country specific subgroups were created to use as many observations available from the dataset; subgroups were created based on their geographic regions delineated by established international organizations such as the United Nations and the World Bank.

Participants’ ages at kindergarten entry ranged from 42.67 months (3 1/2 years) to 88.47 months (7 1/3 years) (*n* = 833, *mean* = 66.03, *median* = 66.23, *SD* = 4.776).

Table 1 lists the demographic characteristics of the sample.

### 2.2. Measures

#### 2.2.1. Spring Science Scores

Participants completed an assessment in the spring of kindergarten. The assessment included 20 questions about physical sciences, life sciences, environmental sciences, and scientific inquiry, consistent with national and state standards. Each child was then assigned a scale score using Item Response Theory (IRT) modeling, with the score being the estimated number of items the child would have answered correctly if given all items in the assessment (Tourangeau et al., 2015).

#### 2.2.2. Opportunity Variables

There were seven opportunity variables: parent-reported child’s bedtime routine (1 if the child goes to bed by 8 pm; 0 if later), rules regarding TV screen time (average of three items, 1 if yes, 0 if no: rules for which programs the child can watch, the number of hours the child may watch TV, and how early or late the child can watch TV), number of structured activities in which the child participates (sum of eight items, 1 if yes, 0 if no: whether the child participates in dance lessons, organized athletic activities, organized clubs, music lessons, drama classes, art classes/lessons, performing arts programs, and craft classes/lessons), number of non-structured activities (sum of seven items, 1 if yes, 0 if no: telling stories, singing songs, helping the child with art, chores, talking about nature, building things, doing sports, playing games), number of “outings” in the past week (sum of six items, 1 if yes, 0 if no: visiting the library, visiting the bookstore, attending a play/concert/show, going to an art museum/history site, visiting a zoo/aquarium/farm, attending a sporting event), how often families read to the child (1 = not at all, 2 = once or twice a week, 3 = three to six times a week, 4 = every day), and how often the child reads to themselves or to others outside of school (1 = not at all, 2 = once or twice a week, 3 = three to six times a week, 4 = every day).

Adequate reliability was demonstrated for multi-item constructs: TV rules (Macdonald’s ω = 0.60), structured activities (ω = 0.68), non-structured activities (ω = 0.69), and outing activities (ω = 0.57).

#### 2.2.3. Propensity Variables

There were six propensity variables: teacher-reported child’s fall math scores, fall reading scores, approach to learning, social skills, and two executive functioning tasks: the Numbers Reversed and the Dimensional Change Card Sort (DCCS).

*Fall Math Scores.* Participants completed a two-stage adaptive standardized assessment in the fall of kindergarten. The two-stage adaptive nature was employed so that children were tested on a set of items according to their demonstrated ability level in the first stage. The assessment tapped their conceptual knowledge, procedural knowledge, and problem solving, consistent with national and state standards. As with the science assessment, an IRT scale score was derived.

*Fall Reading Scores.* Participants completed a two-stage adaptive standardized assessment in the fall of kindergarten. The two-stage adaptive nature was employed so that children were tested on a set of items according to their demonstrated ability level in the first stage. The assessment tapped their basic skills (print familiarity, letter recognition, beginning and ending sounds, rhyming words, and word recognition), vocabulary knowledge, and reading comprehension, consistent with national and state standards. As with the science assessment, an IRT scale score was derived.

*Approaches to Learning.* Teachers reported how often children exhibited six learning behaviors: keeps belongings organized, shows eagerness to learn new things, works independently, easily adapts to changes in routine, persists in completing tasks, pays attention well (6 items total).

*Social Skills.* Teachers completed four separate measures indicating how often children exhibited behaviors related to self-control, interpersonal skills, externalizing problem behaviors, and internalizing problem behaviors (19 items total). Each item was rated on a scale of 1 (never) to 4 (very often). Both the problem behavior scores were reversed so that higher scores in all measures indicated a more positive outcome. Scores on these four measures were averaged.

*Executive Functioning.* Participants completed two tasks: the Numbers Reversed and the DCCS. Scores from these two tasks were standardized and averaged.

*Numbers Reversed.* Participants were asked to repeat a sequence in reverse order (e.g., if a child is presented with 3…5, they say 5…3). Participants started with five two-digit items, and if they had three consecutive items incorrect, they moved on to five three-digit items. The sequence continued to increase in length until the child answered three consecutive items incorrectly. There was a total of 30 trials, including five two-digit number items and four of each of the four- to eight-digit items. Each participant received a *W* score, with a mean of 500 and a standard deviation of 100, and a minimum score of 403 (equivalent to a raw score of 0), which was then standardized.

*DCCS.* Participants were asked to sort a card with a stimulus picture into a tray with a blue rabbit or another tray with a red boat. For instance, when asked to choose by shape, if a red rabbit was presented, they had to place the card in the blue rabbit tray. There were three blocks: first, they were sorted by color (pre-switch block), then by shape (post-switch), and finally by either color or shape, depending on whether the card had a border (the Border Game). In the Border Game, if the card had a border, the child had to sort by color; if it did not, they sorted by shape. Each block contained six trials, and overall accuracy (items correct out of 18) was recorded and standardized.

Reliability for each measure was adequate, according to the ECLS-K manual, as indicated by theta (θ) scores: Approaches to Learning (θ = 0.91), Numbers Reversed (θ > 0.90), DCCS (θ > 0.90), and fall math scores (θ = 0.93). Reliability for social skills, which was the average of four survey data points, was also adequate (ω = 0.68).

#### 2.2.4. Antecedent Variables

There were eight antecedent variables: *family socioeconomic status* (*SES*; a composite variable derived from family income, parental education, and occupational prestige score), *parental expectations* (1 if they expect their child to at least graduate from a four-year college; 0 if lower), *primary language spoken at home* (1 if bilingual; 0 if only one language other than English), *maternal marital status* (1 if married; 0 if not), *number of close grandparents* (0 to 5), *number of siblings* (0 to 9), child gender (1 if “girl”; 0 if not), *poverty level* (1 if below the poverty level), and *immigrant status* (1 if parent or child is “born outside the US”; 0 if not).

### 2.3. Data Analytical Plan

A multi-group path analysis was performed in R using ‘lavaan’ version 0.6.19 (Rosseel, 2012). First we examined the effect of the proposed model, unconstrained, for each subgroup; afterward, we examined the effect of the model at the overall group and subgroup level. Note that although a model with equality constraints is typically done in multi-group analyses, it was not done here as we did not have a hypothesis about which parameters would show subgroup differences; instead, we only show that there may be differences between subgroups. Indirect effects were examined with ‘manymome’ version 0.2.7 (Cheung & Cheung, 2023). In the model, spring kindergarten science scores were regressed on all variables, and opportunity and propensity variables were regressed on antecedent variables. Additionally, the science, math, and reading IRT scores were divided by 10 to allow values for all means of continuous variables to be numerically close to each other. The full information maximum likelihood method used the ‘lavaan::sem’ function to address missing data for the exogeneous variables during the path analysis. MCAR’s Little test indicated that data were missing completely at random (*p* > 0.05).

The data and code that support the findings of this study are available from the corresponding author upon request.

## 3. Results

### 3.1. Are There Differences in Science Performance?

Table 2 presents the means and standard deviations for all variables. Pairwise comparisons of the outcome variable, Spring science scores, indicated that the subgroups were not different from one another (all *p*s > 0.05).

### 3.2. What Factors Predict Science Performance?

Our second research question addressed the factors that predicted diversity of science performance in Asian American children. We fitted a model for the overall group, each subgroup, and a model accounting for subgroups (the multi-group model). In all models, antecedent variables served as predictors and opportunity and propensity variables served as mediators. The model did not fit the data at the overall and subgroup level (Table 3). Modification indices suggest that covarying some variables may improve the fit. These variables include math and reading scores, social skills and approaches to learning, and poverty and SES. These modifications improved the fit of the overall and multi-group models (see bottom rows of Table 3).

Alternatively, the poor fit may be because some of the exogeneous variables were imbalanced; for example, parents who indicated that they would like their children to finish at least college (81%) was disproportionately greater than the those who did not choose that option (3%) or had missing data (16%). Such imbalances might have contributed to larger-than-normal direct effect coefficients (Table 4). Because modifications did not sufficiently improve the fit of the models, the original model was kept; the following results and interpretations warrant caution.

A direct effects analysis revealed that among propensity variables, executive functioning and prior knowledge were positively associated with math scores in the overall group (Table 4, last column). There were significant direct effects of approaches to learning or classroom behaviors for Vietnamese (positive) and Other Southeast Asian (negative) subgroups and executive function (positive) for the Indian subgroup. There were positive effects of math scores for all subgroups except the Other Southeast and Other East groups and reading scores only for the Chinese subgroup. Among opportunity variables, only TV watching routine had a significant effect—for the overall group and the Chinese subgroup. Among antecedent variables, there was a negative direct effect of being born outside the United States (immigrant status; Other Southeast); there were also positive effects of parental expectations (China and Other) and being in poverty (China).

**Figure 2:**
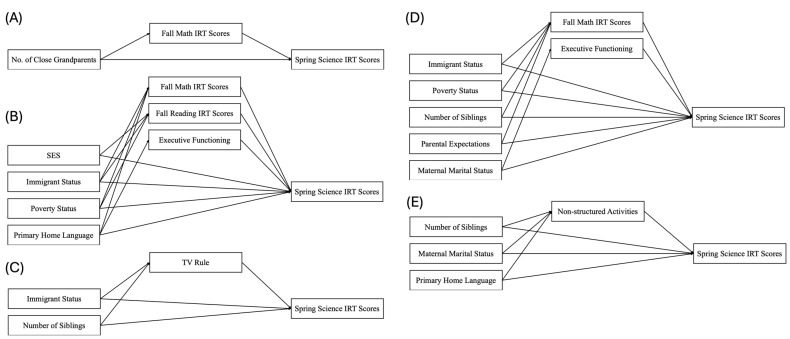
Significant indirect paths for five subgroups: (**A**) Vietnam; (**B**) Other Southeast; (**C**) China; (**D**) India; (**E**) Other.

We also predicted that opportunity and propensity variables would mediate the effect of antecedent variables. There were no significant indirect paths in the overall group model. However, the multi-group analysis showed variation among subgroups in the number of indirect effects (Table 5 and Figure 2). Significant mediators were propensity variables (executive functions and math and reading scores). Non-structured activities and TV-watching routine were the only opportunity variables that significantly mediated antecedent variables.

## 4. Discussion

Emerging research has shown various ways in which cognitive development is shaped by social and cultural factors (e.g., Munakata & Michaelson, 2021; Tsethlikai et al., 2024). Here, we presented an exploratory analysis that examined whether there are differences between subgroups of Asian Americans, both in their science achievement and in how family background and culturally relevant factors predicted their achievement. The results indicate that subgroups were differentially influenced by their immigration status, gender, parental expectations, poverty status, TV-watching routine, self-regulation, and prior knowledge. The antecedent variables of interest—family structure, immigration status, primary home language, and parental expectations—were more likely to have an indirect than direct influence on science scores, and these effects varied by subgroup. Finally, self-regulation skills and prior knowledge most often mediated family background, although home environment (TV-watching routine and non-structured activities) also emerged as mediators.

The study supports findings on the role of self-regulation and prior knowledge. Across the overall group, there were positive effects of executive functions and prior knowledge (reading and math scores), reflecting strong associations among cognitive abilities. This was also the case for all but the Other Southeast and Other East subgroups. This suggests that at kindergarten, self-regulation and knowledge are already strongly related, and that for some children, strengthening self-regulation, math, and/or reading abilities may lead to better science performance. This may be the case especially for Other Southeast Children who are immigrants, as immigrant status negatively predicted science achievement. It is possible that families in this subgroup are socializing their children toward reading and math more so than science, and children are using their reading and math skills for their science abilities. It is unknown whether these effects persist throughout childhood or if children catch up with their other immigrant and non-immigrant peers later in development. Evidence shows that self-regulation and prior knowledge continue to affect children throughout elementary schooling (Spiegel et al., 2021) and that self-regulation itself is bidirectionally related to academic achievement (Cameron et al., 2019; Schmitt et al., 2017). Self-regulation and prior knowledge may also interact such that those with low prior knowledge and high self-regulation could have higher science skills; those with lower achievement at first may also demonstrate compensatory behaviors, such that with high self-regulation, they could catch up with their higher achievement peers (Hernández et al., 2018; A. D. Ribner et al., 2017). However, much of the previous findings have focused on math outcomes; future research should see if these associations apply to the science domain, as well (cf. Byrnes et al., 2018). Promoting self-regulatory behaviors, including through high-quality math instruction (Clements et al., 2016), can be beneficial particularly for those with lower prior knowledge.

In both Vietnamese and Other Southeast Asian children, there was a positive effect of approaches to learning or classroom behaviors for the former group and a negative effect for the latter, providing nuance to previous studies’ findings on the effect of learning behaviors (Bustamante et al., 2018b). It suggests that enhancing classroom behaviors would lead to better outcomes, but this might not necessarily work for Other Southeast Asian children—they might rely instead on social skills, which had a positive (though non-significant) direct effect. A positive indirect effect of primary home language was found for the Other Southeast Asian subgroup through EF and math scores, suggesting speaking English or are bilingual at home also likely boosts this group of children’s science performance. Finally, a negative indirect effect of parental expectations was found through math scores for Indian children: those whose parents had higher expectations likely felt pressured to achieve high math scores, negatively affecting their science performance overall.

Among the surprising findings from this study is the role of TV-watching routines, which was defined here as the existence of rules regarding programs, hours, and timing. It had a direct effect on science performance in the overall group and a mediating role for immigrant status and number of siblings in the Chinese subgroup. Children’s experiences outside of the classroom are important for cultivating interest in and knowledge of science. Such experiences include visiting museums or a zoo (Bradley et al., 2001; Iruka et al., 2014). Watching science TV programs may also be included as a way for learners to engage in science (Liu & Schunn, 2018, 2020). For the overall group and the Chinese subgroup specifically, rules around watching TV negatively affected science scores. It is likely that having fewer restrictions on such activities could positively affect children’s science performance. This may be the case for first- or second-generation immigrants and those with more siblings, as indicated by the significant indirect effects. Other than TV-watching routines, a negative indirect effect of primary home language was found for the Other subgroup through non-structured activities—such activities were negatively associated with science scores, even though being bilingual leads to more non-structured activities.

Altogether, we found various mechanisms in which background and family structures influenced Asian American kindergartners’ science achievement, with most significant mediators being propensity variables: students benefited from their self-regulatory skills and prior knowledge, despite or because of their background. This is consistent with a previous study with the same variables but with math achievement as the outcome (Medrano & Miller-Cotto, 2026). The lower number of direct and indirect paths suggests, however, that what works for improving math outcomes might not work for improving science performance. Nevertheless, the findings reveal how culturally relevant factors may affect science performance—particularly through those propensity and opportunity variables.

### 4.1. Strengths and Limitations

Our paper contributes to our understanding of minority children’s development. Its strengths include the use of a nationally representative dataset, large subgroups of Asian American children, and the number of predictors measured. These factors advance previous studies of Asian American academic outcomes using nationally representative, longitudinal data (e.g., Gibbs et al., 2017; Kao, 1995; Hsin & Xie, 2014) by integrating recent findings in the child development literature on the role of self-regulation, including executive functions, working memory, and approaches to learning, among others (Miller-Cotto et al., 2024; Vitiello & Greenfield, 2017) in science learning; by applying a specific framework that integrates several theoretical models (e.g., family investment and family stress models); and by considering culturally relevant factors.

Our study is an exploration of how culturally relevant factors may affect achievement through child characteristics and home environment. Our analyses confirm and expand on previous findings. Based on previous studies, gender, SES and poverty status should correlate with executive functions and achievement scores and explain some gaps in those outcomes (Byrnes et al., 2018; Cimpian et al., 2016; Conway et al., 2018; Little, 2017; Reardon & Portilla, 2016). We found these to be reflected at the subgroup but not overall level. For Other Southeast Asian children, we found a positive indirect effect of both SES and poverty through math and reading scores; for Indian children, we found a positive effect of poverty through math scores. These findings extend previous studies on socioeconomic gaps in science achievement by accounting for racial group (Barnes et al., 2024; Betancur et al., 2018).

Our study is not without its limitations. While a nationally representative dataset enabled us to examine nuances in Asian American child development, we lacked sufficient data to adequately represent other groups, such as Filipino, South Korean, and Japanese Americans, each comprising fewer than 100 children. This might be because children from these families have resided in the U.S. for generations—their parents may have been born in the U.S., and/or their primary home language is English. In our data, there were 61 children born in the U.S. or children whose parents were born in the U.S. Additionally, first- and second-generation immigrants were possibly overrepresented, with 83% being born outside the U.S. These generations tend to outperform third- and later-generation immigrants on standardized tests (Duong et al., 2016). Finally, it is uncertain whether subgroups were equally represented geographically in the U.S. Future research could address these issues regarding representation.

Relatedly, the analyses may not be as adequate for available data, as in previous studies, due to the sample being reduced to one racial group. Thus, the ratio of parameter to sample size for the full model and for each subgroup model may have not been sufficient; this also probably contributed to model fit to be inadequate. With computational limitations, it is possible that indirect effects were not estimated correctly, and with multiple testing, the risk of a Type I error or a false positive cannot be entirely ruled out. Some of the coefficient values were greater than 1 (e.g., the direct effects of parental expectations and immigrant status in Chinese American children), which may indicate that multicollinearity or a non-linear relationship exists. In the case of Chinese American children, parents tended to expect their child to finish college or more (*n* = 118) with only one indicating less than college and five not indicating a response; most parents and/or children indicated being immigrants (*n* = 159), with others indicating being born in the U.S. (*n* = 1) or not responding (*n* = 14). Thus, for the Chinese subgroup, parental expectations and immigrant status may not be good predictors of science achievement. As we also noted in our data analysis section, we only examined the unconstrained model and that we did not test for specific subgroup differences on certain parameters. More research, including secondary data analyses of larger, existing data as well as research that explores the links between proposed variables, need to be done in order to reach accurate conclusions. Future studies should intentionally examine the effects of these demographic characteristics.

It is possible that the model fitted poorly because the assessments weren’t created with persons of color in mind; thus, the variables that would likely produce good fit are unlikely to exist in such a dataset. For example, in an analysis of the same longitudinal dataset, Miller-Cotto et al. (2026) found that a model with three EF measures (DCCS and Numbers Reversed as in the current study, and a flankers task measuring inhibitory control) converging into one factor was a good fit for subsamples of White, Latine, and Asian, but not Black, students. When examining only those three groups, no two groups fit the model in the same way, suggesting that the tasks are likely measuring different constructs. Multiple possibilities may explain this noninvariance, including differences in how subgroups are socialized in the U.S. This further denotes the importance of examining how culture may affect how individuals respond in assessments (Miller-Cotto et al., 2022; Gaskins & Alcalá, 2023).

Finally, the model was also only one of the models that have been tested in previous studies. That is, we did not test whether the propensity factors also mediated the effect of opportunity factors (Byrnes et al., 2018; Lewis & Farkas, 2017). For example, structured activities might positively relate to social skills and self-regulation because children learn to (positively) interact with at least one other person and to concentrate on the tasks during those activities. Identifying these mechanisms is an important endeavor because of its theoretical and practical implications.

### 4.2. Implications for Theory and Practice

We propose that cultural values are weakly related to achievement, inasmuch as cultural values are often too global, or distal to achievements. A better model would posit that cultural values or socialization patterns affect a mediator (a more proximal variable such as effort or motivation), which is likely to show a stronger correlation with achievements. The mediator is also influenced by other variables, besides culture, such as opportunities for advancement in other areas of life.” (Sue & Okazaki, 2009, p. 50)

As in Medrano and Miller-Cotto (2026), the O-P Model was used to conceptualize the role of Asian American children’s background, prior knowledge, self-regulation, and various resources in their science achievement. Following previous studies, we added variables that may be important in our sample, such as immigration status and number of close grandparents and siblings. We support the use of this framework to investigate the links between important variables in other scenarios. Future studies could include other variables, such as those related to neighborhood characteristics and cognitive stimulation, as those could also mediate the role of family characteristics on performance (Dearing et al., 2009).

An implication of the findings above is that it would be difficult to design an intervention that could benefit all children. A program might focus on strengthening children’s self-regulation abilities, social skills, and prior knowledge, as they predicted most of the children’s science performance. Families and educators may teach children to resist distractions or redirect their attention to the task at hand, encourage them how to work with others, and engage their prior knowledge. Science curricula could integrate math and literacy content and emphasize the importance of both skills for scientific thinking. For example, an instructor could teach physical concepts like gravity and mass while also teaching mathematical vocabulary like “more” or “less” and the process of inference and cause and effect. Early science learning researchers have designed comprehensive interventions that could also be adopted more broadly (Chen et al., 2024; Whittaker et al., 2020). At home, families would benefit from learning more about the connections between math, reading, and science skills and information on how to engage in science activities outside of the classroom or before formal schooling, whether it’s watching science TV programs or participating in informal learning experiences such as visiting museums. Such activities may affect not only their science knowledge but their interest and motivation in doing science.

One policy implication is further disaggregation of data. Disaggregating data by subgroup membership, for example, could inform targeted interventions. Better reporting and knowledge of the data may also help address important issues that would otherwise be left unaddressed. As it has been mentioned, wide inequalities exist in some subgroups (Chinese, Japanese, and Korean); it would be unfair to group all these students and assume that everyone is performing similarly. Such disaggregation will lead toward more attention not only to racial subgroups but also economic status and experiences (e.g., refugees, first-generation immigrants, socioeconomically disadvantaged, and English language learners).

## 5. Conclusions

Perhaps the most important lesson to be learned from this study is that not all Asian American children are affected in the same way by their family and socioeconomic background, perhaps owing to their background differences historically, economically, culturally, and socially. A theoretical perspective that considers these factors, as a result, also has practical implications that are not necessarily universal but are more realistically sensitive to various contexts.

## Figures and Tables

**Table 1:** Demographic Characteristics.

Variable and Levels	Frequency	%
***N***	894	100
**Subgroup**		
Vietnam	121	13.5
Other Southeast	135	15
China	174	19.5
Other East	106	12
India	189	21
Other	169	19
**Gender**		
(1) Female	479	54
(0) Male	415	46
**Poverty Level**		
(1) Below Threshold	144	16
(0) Above Threshold	690	77
No Data	60	7
**Maternal Marital Status at Child’s Birth**		
(1) Married	726	81
(0) Single	104	12
No Data	64	7
**Primary Home Language**		
(1) English or Bilingual	390	44
(0) Only Non-English Language	500	56
No Data	4	<1
**Parent Expectations to Finish College**		
(1) Finish College	722	81
(0) Less than College	29	3
No Data	143	16
**Immigrant Status**		
(1) Child or Parent Born Outside the US	745	83
(0) Child or Parent Born In the US	85	10
No Data	63	7

**Table 2:** Means and Standard Deviations for All Variables.

	Vietnam	Other Southeast	China	Other East	India	Other	Overall
*n*	121	135	174	106	189	169	894
Spring Science IRT Scores	3.41 (0.75)	3.26 (0.72)	3.34 (0.79)	3.36 (0.69)	3.39 (0.72)	3.43 (0.78)	3.37 (0.75)
Propensity Factors							
Social Skills	3.28 (0.51)	3.28 (0.46)	3.28 (0.5)	3.3 (0.49)	3.31 (0.47)	3.25 (0.51)	3.29 (0.49)
Approaches to Learning	2.96 (0.74)	2.91 (0.65)	3.01 (0.7)	2.96 (0.6)	3.04 (0.64)	2.9 (0.71)	2.97 (0.68)
Executive Functioning	0.04 (0.7)	−0.07 (0.81)	−0.05 (0.88)	0.04 (0.69)	0.07 (0.82)	−0.03 (0.83)	0 (0.8)
Fall Math IRT Scores	3.62 (1.15)	3.44 (1.14)	3.61 (1.22)	3.53 (0.95)	3.65 (1.16)	3.66 (1.24)	3.59 (1.16)
Fall Reading IRT Scores	5.47 (0.93)	5.28 (1.08)	5.59 (1.41)	5.38 (0.96)	5.46 (1.31)	5.61 (1.44)	5.48 (1.24)
Opportunity Factors							
Nonstructured activities	1.88 (1.3)	2.57 (1.29)	2.14 (1.31)	2.79 (1.12)	2.34 (1.15)	2.56 (1.24)	2.39 (1.26)
Structured activities	0.78 (1.01)	0.85 (1.21)	1.52 (1.43)	1.71 (1.4)	1.76 (1.56)	1.18 (1.28)	1.33 (1.39)
TV rule	0.76 (0.34)	0.9 (0.23)	0.81 (0.3)	0.85 (0.25)	0.89 (0.22)	0.86 (0.23)	0.85 (0.26)
Bedtime rule	0.22 (0.42)	0.38 (0.49)	0.31 (0.46)	0.51 (0.5)	0.47 (0.5)	0.58 (0.5)	0.42 (0.49)
Parent reading	2.86 (0.81)	3.12 (0.74)	3.11 (0.74)	3.28 (0.64)	3.21 (0.68)	3.15 (0.78)	3.13 (0.74)
Child reading	2.94 (0.91)	3.13 (0.81)	3.31 (0.8)	3.52 (0.65)	3.29 (0.75)	3.21 (0.87)	3.24 (0.81)
Outings	2.59 (1.77)	3.24 (1.66)	2.8 (1.53)	3.57 (1.49)	3.02 (1.41)	2.92 (1.62)	3 (1.59)
Antecedent Factors							
Gender	0.5 (0.5)	0.52 (0.5)	0.59 (0.49)	0.48 (0.5)	0.56 (0.5)	0.54 (0.5)	0.54 (0.5)
Immigrant Status	0.03 (0.17)	0.06 (0.23)	0.01 (0.08)	0.09 (0.28)	0.02 (0.15)	0.37 (0.48)	0.1 (0.3)
Parental Expectations	0.94 (0.24)	0.94 (0.23)	0.99 (0.09)	0.96 (0.2)	0.98 (0.13)	0.94 (0.23)	0.96 (0.19)
Number of Siblings	1.24 (0.92)	1.23 (1.04)	1.07 (0.87)	1.08 (0.72)	0.96 (0.7)	1.56 (1.16)	1.2 (0.94)
No. of Close Grandparents	1.96 (1.18)	1.78 (1.24)	2.37 (1.28)	2.49 (1.21)	2.28 (1.24)	1.99 (1.25)	2.14 (1.26)
Maternal Marital Status	0.82 (0.39)	0.8 (0.4)	0.89 (0.32)	0.88 (0.33)	0.99 (0.1)	0.83 (0.38)	0.87 (0.33)
Primary Home Lang.	0.2 (0.4)	0.66 (0.48)	0.26 (0.44)	0.48 (0.5)	0.36 (0.48)	0.69 (0.46)	0.44 (0.5)
Poverty status	0.31 (0.47)	0.19 (0.39)	0.21 (0.41)	0.08 (0.27)	0.03 (0.18)	0.23 (0.43)	0.17 (0.38)
SES	−0.28 (0.77)	0.24 (0.73)	0.29 (1)	0.82 (0.68)	0.93 (0.72)	0.18 (0.81)	0.38 (0.89)

SES = Socioeconomic Status; IRT = Item Response Theory.

**Table 3:** Fit indices.

Group	X^2^	df	*p*	CFI	RMSEA [90% CI]	SRMR
Vietnam	488.51	102	<0.001	0.230	0.177 [0.161, 0.193]	0.134
Other Southeast	497.92	102	<0.001	0.224	0.170 [0.155, 0.185]	0.129
China	508.865	102	<0.001	0.388	0.151 [0.138, 0.165]	0.143
Other East	342.855	102	<0.001	0.189	0.149 [0.132, 0.167]	0.113
India	487.364	102	<0.001	0.304	0.141 [0.120, 0.154]	0.108
Other	651.024	102	<0.001	0.164	0.178 [0.166, 0.192]	0.133
Overall	2554.519	102	<0.001	0.252	0.164 [0.159, 0.170]	0.120
Multi-group	2976.537	612	<0.001	0.258	0.161 [0.155, 0.167]	0.127
Overall (modified)	636.11	95	<0.001	0.835	0.080 [0.074, 0.086]	0.073
Multi-group (modified)	1144.12	570	<0.001	0.820	0.082 [0.075, 0.089]	0.089

Note. For the modified models, the following pairs of variables were covaried: math and reading IRT scores, social skills and approaches to learning, and poverty and SES.

**Table 4:** Coefficients of Direct Effects on Spring Science Scores (Standardized).

	Vietnam	Other S.E.	China	Other East	India	Other	Overall
Propensity Factors							
Social Skills	−0.303	0.379	−0.015	0.017	−0.132	0.182	−0.053
Approaches to Learning	**0.317**	**−0.324**	0.072	0.008	0.141	−0.092	0.095
Executive Functioning	0.178	0.166	0.138	0.097	**0.176**	0.102	**0.149**
Fall Math IRT Scores	**0.211**	0.197	**0.269**	0.183	**0.311**	**0.306**	**0.259**
Fall Reading IRT Scores	0.108	0.115	**0.167**	0.186	0.007	0.037	**0.061**
Opportunity Factors							
Nonstructured activities	−0.06	0.014	0.071	0.027	0.08	−0.11	−0.001
Structured activities	−0.072	0.032	0.007	−0.021	−0.035	0.008	−0.013
TV rule	0.023	−0.107	**−0.494**	0.011	0.077	−0.033	**−0.181**
Bedtime rule	0.079	0.087	−0.218	0.032	−0.071	0.022	0.012
Parent reading	−0.082	−0.025	0.044	−0.011	0.078	0.116	0.032
Child reading	0.017	0.017	−0.056	−0.095	−0.05	0.009	−0.015
Outings	−0.072	−0.072	0.041	−0.006	0.021	−0.035	−0.007
Antecedent Factors							
Gender	**0.274**	−0.101	0.166	−0.01	−0.034	−0.038	0.04
Immigrant Status	−0.182	**−0.506**	1.18	0.229	−0.249	0.062	0.02
Parental Expectations	0.032	0.189	**4.331**	−0.305	−0.513	**0.418**	0.17
Number of Siblings	−0.097	0.039	0.09	−0.024	−0.04	0.009	0.009
No. of Close Grandparents	0.006	0.026	0.063	−0.077	−0.03	0.053	0
Maternal Marital Status	0.082	0.025	0.144	0.158	0.078	−0.147	−0.006
Primary Home Language	−0.064	0.154	0.197	−0.073	−0.127	−0.05	0
Poverty status	0.013	0.268	**0.313**	−0.263	0.129	0.117	0.069
SES	0.036	0.019	−0.002	0.077	0.079	0.131	0.044

Bolded values indicate significant values (*p* < 0.05). SES = Socioeconomic Status; IRT = Item Response Theory.

**Table 5:** Significant Indirect Effects (Unstandardized).

Paths	Indirect Effect, 95%CI
Vietnam	
Number of Close Grandparents -> Fall Math IRT Scores	−0.046 [−0.167, −0.012]
Other Southeast	
Immigrant Status -> Fall Math IRT Scores	0.221 [0.014, 0.563]
Immigrant Status -> Fall Reading IRT Scores	0.168 [0.018, 0.286]
Poverty Status -> Fall Math IRT Scores	0.166 [0.081, 0.421]
Poverty Status -> Fall Reading IRT Scores	0.101 [0.010, 0.293]
Primary Home Language -> Executive Functioning	0.042 [0.006, 0.162]
Primary Home Language -> Fall Math IRT Scores	0.054 [0.018, 0.201]
SES -> Fall Reading Scores	0.047 [0.014, 0.098]
China	
Immigrant Status -> TV Rule	−0.085 [−0.162, −0.036]
Number of Siblings -> TV Rule	−0.020 [−0.053, −0.002]
India	
Immigrant Status -> Fall Math IRT Scores	−0.314 [−0.527, −0.192]
Poverty Status -> Fall Math IRT Scores	0.225 [0.026, 0.512]
Number of Siblings -> Fall Math IRT Scores	−0.061 [−0.136, −0.004]
Maternal Marital Status -> Executive Functioning	−0.098 [−0.226, −0.016]
Parental Expectations -> Fall Math IRT Scores	−0.268 [−0.480, −0.066]
Other	
Number of Siblings -> Non-structured Activities	−0.024 [−0.065, −0.004]
Maternal Marital Status -> Non-structured Activities	0.090 [0.008, 0.221]
Primary Home Language -> Non-structured Activities	−0.054 [−0.116, −0.001]

Due to computational limitations, the indirect effects for the multi-group analysis were bootstrapped from 17 bootstrap samples; overall is from 100 samples. SES = Socioeconomic Status; IRT = Item Response Theory.

## Data Availability

No new data were created or analyzed in this study.
